# The effect of evening primrose oil on cervical ripening and birth outcomes: A systematic review and meta-analysis

**DOI:** 10.1016/j.heliyon.2023.e13414

**Published:** 2023-02-08

**Authors:** Sholeh Shahinfar, Parvin Abedi, Shayesteh Jahanfar, Mahin Khajehpoor, Mohammadreza Chashmyazdan

**Affiliations:** a Midwifery Department, Faculty of Nursing and Midwifery, Kerman Branch, Islamic Azad University, Kerman, Iran; b Menopause Andropause Research Center, Ahvaz Jundishapur University of Medical Sciences, Ahvaz, Iran; c MPH Program, Department of Public Health and Community Medicine, Tufts University School of Medicine, USA; d Noncommunicable Diseases Research Center, Bam University of Medical Sciences, Bam, Iran; e Kerman University of Medical Sciences, Kerman, Iran

**Keywords:** Bishop score, Cervical ripening, Evening primrose oil, Systematic review

## Abstract

**Background and purpose:**

The results of various studies on the effect of evening primrose oil (EPO) on cervical ripening are controversial. The purpose of this systematic review and meta-analysis was to assess the effect of EPO on cervical ripening and birth outcomes.

**Materials and methods:**

The Cochrane Library, Embase, PubMed, Scopus, Web of Science and Persian databases were searched for studies published from the inception of the databases up to February 2021 (search updated in May 2022). Full-text articles published in English or other languages, randomized controlled trials, and quasi experimental studies with control group were included. Studies published in form of conference proceedings, and those whose full texts were not available, as well as studies with control groups receiving other treatments for cervical ripening, and those in which the intervention group received drugs besides EPO were all excluded. The Cochrane handbook was used to determine the risk of bias of the included studies. All data were analyzed using Review Manager 5.4 and reported in forest plots.

**Results:**

Seven trials involving 920 women were included in the meta-analysis. In five studies, including 652 participants, cervical ripening was evaluated using Bishop score. The use of EPO was found to significantly improved Bishop score (MD = 3.23; 95% CI: 3.17, 3.29). The meta-analysis showed no significant differences between two comparison groups in terms of 1-min Apgar score and length of the second stage of labor. However, the two groups were significantly different in terms of their 5-min Apgar score and the time interval between administration of EPO and birth. Based on subgroup analysis by route of administration, both vaginal and oral use of EPO increased Bishop score significantly in the intervention group compared to the placebo group.

**Conclusion:**

This study showed that using EPO in term and post-term pregnant women was clinically effective in improving their Bishop score.

## 1 Introduction

The progressive process of cervical ripening commences in the first trimester of pregnancy and continues until term [1]. An unripe cervix increases the risk of caesarean section and prolonged labor [2]. Prolonged duration of second stage has been associated with some maternal and neonatal morbidities such as third and fourth-degree tears, post-partum hemorrhage, low Apgar scores and sepsis [3]. In high-risk pregnancies, when the continuation of pregnancy is dangerous for the mother and baby, induction of labor (IOL) is one of the measures taken to reduce maternal and infant mortality [4,5]. Cervical ripening is the most important factor in the success of IOL and increases the likelihood of vaginal birth [6,7]. The pre-induction ‘favorability’ of the cervix is assessed by the Bishop score, which is a standard method for the prediction of the duration and outcome of induced labor [8]. The Bishop Scoring system was first described by Bishop in 1964 and is based on assessing multiple items (the position, consistency, effacement, and dilatation of the cervix, and the fetal station) [9]. A score below six is indicative of a reduced success [3] of vaginal birth [10]. Numerous approaches have so far been suggested for cervical ripening and labor induction, which are divided in two groups of mechanical and medical. Mechanical methods include a *trans*-cervical catheter, hygroscopic dilators and stripping, whereas medical methods consist of some agents such as prostaglandins E1 and E2. Using mechanical methods may lead to increased caesarean sections, uterine infections and unnecessary vaginal examinations [11]. Misoprostol (prostaglandin E1), which is widely used for induction of labor, is associated with maternal and fetal complications such as diarrhea, abdominal pain, hyperthermia and fetal tachysystole [[12], [13], [14]]. Hence, given the maternal and neonatal adverse effects associated with pharmaceutical induction methods, there has always been a concern for midwives and obstetricians in achieving effective low-risk interventions [9].

In addition to the methods described above, there are some traditional methods such as different forms of herbal medicines, which are used for preparing the cervix and accelerating childbirth [11]. Using such medicines for cervical ripening is accompanied with many benefits, such as decreased number of post-term pregnancies, and less need for elective induction, and increased rate of successful induction [15]. An example of such herbs is evening primrose oil (EPO), which is used by some health providers for cervical ripening and labor initiation [16]. EPO is recommended in some midwifery literatures as a cure for post-dates pregnancy, and more than 60% of US nurse-midwives prescribe it in late pregnancy [17,18]. It is also the most common herbal remedy for cervical ripening used by certified midwives in Iran [19].

Evening primrose, scientifically known as Oenothera Biennis belongs to the Onagraceae family [20]. In addition to its use as a treatment for some inflammatory diseases such as rheumatoid arthritis, evening primrose is used for managing women's health issues, including mastalgia, premenstrual and menopausal symptoms, and labor induction or augmentation [21]. The therapeutic effect of primrose is attributed to the oil extracted from its seeds, which comprises mainly of linoleic acid (60%–65%), gamma-linolenic acid (7%–14%), oleic, palmitic, and stearic acids [22,23]. Although EPO is a product most frequently used by health providers, its effectiveness in cervical preparation and shortening the duration of labor has been a controversial topic in literature. According to a quasi-experimental study by Dove et al., oral administration of EPO from 37 weeks of gestation till birth, had no effect on reducing the duration of pregnancy or length of labor [17]. Investigating the effectiveness of EPO vaginal suppositories on cervical readiness of term pregnancy, Nonette et al., found a positive effect of EPO on Bishop score [24]. Also, a randomized controlled trial on 71 pregnant women with full-term pregnancies showed that daily consumption of three oral capsules of EPO for one week could increase the Bishop score and cervical effacement [25]. The greater number of reviewed articles and outcomes are the distinguishing feature of this review from the recent systematic reviews. Furthermore with different results of two prior systematic reviews, we cannot draw a definite conclusion about the effect of EPO on cervical ripening. Therefore, this systematic review and meta-analysis was designed with the aim of assessing the effect of EPO on cervical ripening and birth outcomes.

## 2 Materials and Methods

### 2.1 Study registration

This systematic review and meta-analysis followed the methodology consistent with Preferred Reporting Items for Systematic Reviews and Meta-Analyses (PRISMA2020) (Supplementary material). The protocol of this systematic review and meta-analysis was registered in PROSPERO with the reference number of CRD42021230488.

### 2.2 Search strategies

We searched for published studies in the following databases: The Cochrane Central Register of Controlled Trials (CENTRAL), Embase, PubMed, Medline, Scopus, Web of Science and Persian databases (Magiran, SID, IRCT.ir) from database inception up to March 2021 (search was updated in May 2022). Some of the search keywords used were: “evening primrose oil” AND “Bishop score” OR “cervical ripening” OR “cervix preparation” OR “labor induction” AND “prolonged pregnancy”.

### 2.3 Inclusion and exclusion criteria

#### 2.3.1 Types of included studies

We included the following studies: a) full-text articles published in English or other languages, b) randomized controlled trials, c) quasi experimental studies with control groups. Studies published in form of conference proceedings and those whose full texts were not available, as well as studies with control groups receiving other treatments for cervical ripening, and those in which the intervention group received drugs besides EPO were all excluded. We also excluded studies on the effect of EPO on other diseases.

#### 2.3.2 Types of participants

Women who were nullipara with term or post term pregnancy were included.

#### 2.3.3 Types of intervention

##### 2.3.3.1 Experimental intervention

Studies using EPO alone were included.

##### 2.3.3.2 Control intervention

Studies in which the control groups received placebo were included.

### 2.4 Types of outcome measures

#### 2.4.1 Primary outcomes

Improvement of Bishop Score
Length of the second stage of labor
The time interval between primrose administration and childbirth

#### 2.4.2 Secondary outcomes

One and 5-min Apgar scores
Adverse effects of EPO administration

### 2.5 Data collection and quality assessment

#### 2.5.1 Literature selection and data extraction

The search was carried out by SS and MC. Two review authors (SS and MK) independently screened titles and abstracts of all searched studies as well as their full texts using the Covidence software. Possible conflicts between authors, were resolved by discussion. When two reviewers were unable to reach a consensus, the conflict was resolved by a third party (PA or SJ). For data extraction, we designed a form according to the data extraction form recommended by the Pregnancy and Childbirth Cochrane Group. Two review authors (SS and MK) independently extracted information from the included studies. The following pieces of information were extracted: study design (method), participants, intervention, risk of bias, inclusion and exclusion criteria, Bishop score, length of labor, and outcomes of the pregnancy.

#### 2.5.2 Assessment of risk of bias and quality of the included studies

Two review authors (SS and MK) used the Cochrane Collaboration's Tool for assessing risk of bias in randomized trials. They assessed the risk of bias for each study independently and in cases of disagreement, they consulted a third person (PA). The following domains of risk of bias (ROB) were assessed for each trial: random sequence generation (selection bias), allocation concealment (selection bias), blinding of participants and personnel (performance bias), blinding of outcome assessment (detection bias), incomplete outcome data (attrition bias), selective reporting (reporting bias), and other biases. Each potential risk of bias was graded as high, low, or unclear.

Studies were qualified using GRADE (The Grades of Recommendation, Assessment, Development and Evaluation Working Group). Table 2.

### 2.6 Statistical analysis

#### 2.6.1 Strategy for data synthesis

Mean differences (MD) with 95% confidence intervals (95% CI) were calculated to assess the differences between intervention and control groups in terms of Bishop scores, length of the second stage of labor, the time interval between primrose administration and birth, and Apgar score one and 5 min after birth. Forest plots were used to demonstrate effect sizes and 95% CI. Heterogeneity among the included studies was assessed by I^2^ statistics. By default, we used a fixed-effect model for all pooled studies.

According to the primary results of heterogeneity, if I^2^>40%, a random-effect model was used to be compared with the fixed-effect model. We also conducted a sensitivity analysis to explore the potential sources of heterogeneity if and when the heterogeneity across studies was statistically significant. Sensitivity analyses were carried out by sequentially omitting one single study each time to test the robustness of uncertainty in the meta-analysis. All data were analyzed using Review Manager (Rev- Man 5.4). The significance level was set ≤0.05.

We carried out sub-group analysis by route of administration (vaginal versus oral rout of administration).

#### 2.6.2 Dealing with missing data

After gathering missing data, incomplete information, or data errors, we asked the corresponding author via email or telephone for the correct information.

## 3 Results

### 3.1 Literature search

**Fig. 1:**
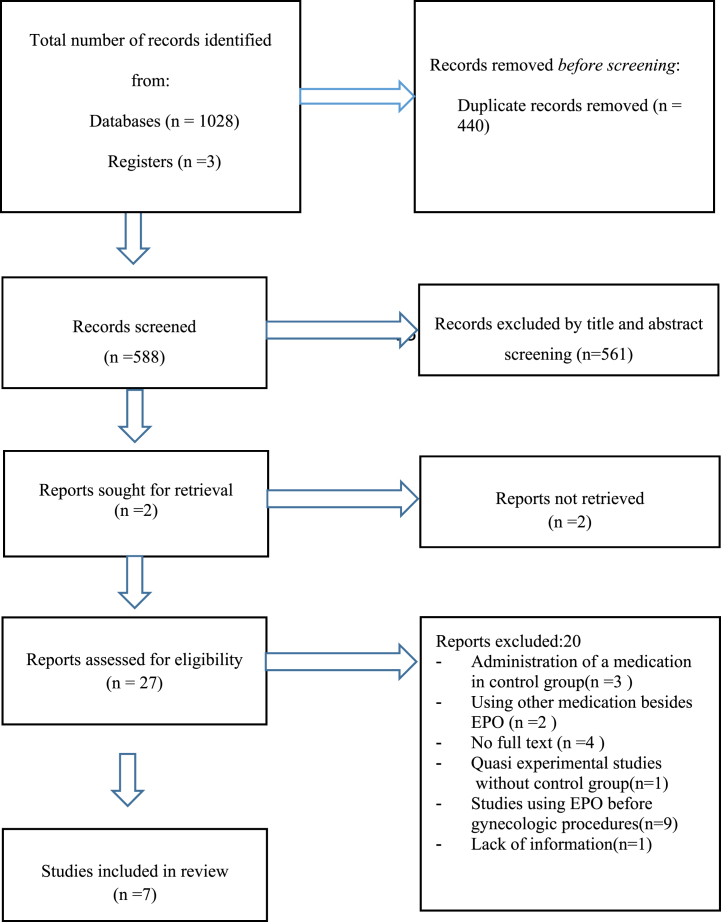
PRISMA flow diagram of article selection progress.

A flow diagram of the included and excluded studies is shown in Fig. 1. The database searches identified 1028 records. After removing the duplicates (n = 440), two reviewers (SS and MK) independently screened the titles and abstracts for potentially relevant studies (n = 588), and 561 studies were not relevant to the review. 26 full-text articles were considered eligible. 20 studies were excluded, so six studies were finally included in the meta-analysis. Among 20 excluded study, in three studies a medication (misoprostol, castor oil, oxytocin) were administered in control groups, in two studies other medications besides EPO were used, the full text of four studies were not accessible and after contacting the authors, we did not get any response, a quasi-experimental study without control group, In nine study EPO was used before gynecologic procedure in non-pregnant women, one study was without enough information. We updated the search in May 2022 and two articles were added, but one of them was excluded, because it involved a different treatment for cervical ripening (misoprostole) in the control group [26]. Therefore, seven studies were considered for meta-analysis.

### 3.2 Characteristics of the included studies

The seven included studies were published from 1999 to 2022. Six studies were randomized controlled trials (RCTs) and one was quasi-experimental study with a control group. Four studies were conducted in Iran [10,19,27,28], and three were carried out in Egypt, the United States, and the Philippines [15,17,25]. The studies included a total of 920 participants. The characteristics of the studies included in meta-analysis are shown in Table 1. The sample size of the mentioned studies ranged from 71 to 240. As far as cervical preparation was concerned, five studies have investigated the Bishop score and birth outcomes [15,19,27,28], and one measured only the time span between the administration of EPO and onset of childbirth [10].

**Table 1:** Characteristics of included studies.

Reference	Methods	Participants	Interventions (Dosage)	Interventions (Duration of treatment)	Outcomes
Azad et al., 2022	RCT	EG:88	EG: 500 mg vaginal EPO capsules	6 h before oxytocin induction of labor	-Bishop score
	-double blind	CG^:^ 87	CG: Placebo vaginal		-Duration of the latent phase
	-two arms		6 h before oxytocin induction of labor		- The parturition time
					- length of the active phase
					-The second stage of labor
					-Apgar scores
Hashemnejad et al., 2019	-RCT	EG:80	EG: 2 vaginal capsule EPO 1000	From 37 weeks gestation to contractions and birth onset	-The time interval between intervention and contractions initiation
	-triple blind	CG:80	CG: 2 placebo vaginal capsule		-The time span between intervention and birth onset
	-two arms				**-** Cesarean rate
Najafi et al., 2019	-RCT	EG:43	EG: vaginal capsule EPO 1000 daily	From 38 weeks gestation until birth	-Bishop score
	-double blind	CG:43	CG: Vaginal capsule		-Duration of the latent, active and second stage of labor
	-two arms		Placebo daily		-Induction and augmentation of labor
					- First and fifth minute Apgar scores
					-Mode of delivery
					-Postpartum hemorrhage
Kalati et al., 2018	-RCT	EG:40	EG: oral capsule EPO 1000 two times daily	40 weeks gestation (7 days)	-Bishop score
	-triple blind	CG:40	CG: oral capsule placebo two times daily		-Duration of pregnancy and labor phases
	-two arms				
Zahran 2009	-RCT	EG:120	EG: oral capsule EPO 1000 two times daily	10 days	-The frequency of successful induction
	-double blind	CG:120	CG: oral capsule placebo two times daily		-Bishop score
	-two arms				**-** Cesarean rate
					-The time from first dose to active labor
					-Induction-to-delivery interval
					-Duration of labor
					-Neonatal outcomes
Ty-torredes et al., 2006	-RCT^a^	EG:38	EG: oral capsule EPO 1500 mg three times daily	Full-term pregnancy (1 week)	-Bishop score
	-Double blind	CG:33	CG: oral capsule placebo three times daily		
	-two arm				
Dove et al., 1999	-RCT^a^	EG^b^:54	EG: Oral EPO capsule three times daily	1 week	-length of labor
	-Double blind	CG^c^:54	CG: Oral EPO^d^ capsule placebo three times daily		-Incidence of postdate induction, -Prolonged ROM
	-two arms				-Cesarean, vacuum, NVD in 14 days
					-Apgar scores
					-Neonatal body weight

a RCT = Randomized Controlled Trial.

b EG: Experimental group.

c CG: Control group.

d EPO = Evening Primrose Oil.

**Table 2:** Quality assessment.

Outcomes	Anticipated absolute effects* (95% CI)		№ of participants (studies)	Certainty of the evidence (GRADE)	Comments
	Risk with Placebo	Risk with Evening primrose			
Bishop score	The mean bishop score ranged from **3**–**7**	MD **1.8 higher** (0.32 higher to 3.28 higher)	652 (5 RCTs)	⨁⨁⨁◯ Moderate^a^	
Bishop score (vaginal)	The mean bishop score (vaginal) ranged from **3**–**4**	MD **3.29 higher** (3.23 higher to 3.35 higher)	261 (2 RCTs)	⨁⨁⨁⨁ High	
Bishop score (oral)	The mean bishop score (oral) ranged from **4**–**7**	MD **0.77 higher** (0.58 lower to 2.13 higher)	391 (3 RCTs)	⨁⨁⨁⨁ High	
The second stage of labor	The mean the second stage of labor ranged from **50**–**70** Minutes	MD **3.97 Minutes lower** (9.33 lower to 1.4 higher)	341 (3 RCTs)	⨁⨁⨁⨁ High	
The time interval between EPO use till childbirth	The mean the time interval between EPO use till childbirth ranged from **2**–**8** Minutes	MD **0.21 Minutes lower** (1.17 lower to 0.75 higher)	482 (3 RCTs)	⨁⨁⨁⨁ High	
One-minute Apgar score	The mean 1-min Apgar score ranged from **8**–**9**	MD **0.08 lower** (0.22 lower to 0.05 higher)	449 (4 RCTs)	⨁⨁◯◯ Low^b^	
Five-minute Apgar score	The mean 5-min Apgar score ranged from **9**–**10**	MD **0.32 higher** (0.24 lower to 0.89 higher)	449 (4 RCTs)	⨁⨁◯◯ Low^b^	

EPO was used in form of vaginal suppository in three trials [10,19,28]. In one study, a daily dose of 1000 mg vaginal suppository of EPO from the 38th week of pregnancy until birth was used [28]. In Hashemnejad et al.’s (2019) study, a single dose of 1000 mg vaginal suppository of EPO was used at 37 weeks of pregnancy [10]. In another study, two soft capsules containing 500 mg of EPO, was applied 6 h before induction of labor with oxytocin [19]. Oral capsules of EPO were used in four trials [17,25,27,29]. In one of the included studies, 1000 mg oral capsule was administered, twice daily, for 7 days from the 40th week of pregnancy until childbirth [27]. In Zahran et al.’s (2009) study, participants attending the antenatal care clinic, received 1000 μg of oral EPO capsules every 12 h for 10 days [29]. In one study, 1500 mg oral EPO capsules were used three times daily for one week, and in another study, the prescribed dosage of EPO was 500 mg orally three times daily for seven days, beginning at the 37th weeks of gestation, and then was reduced to 500 mg orally once per day until labor [17,25]. In all studies, Bishop score was used for assessment of cervical ripening.

### 3.3 Risk of bias in the included studies

**Fig. 2:**
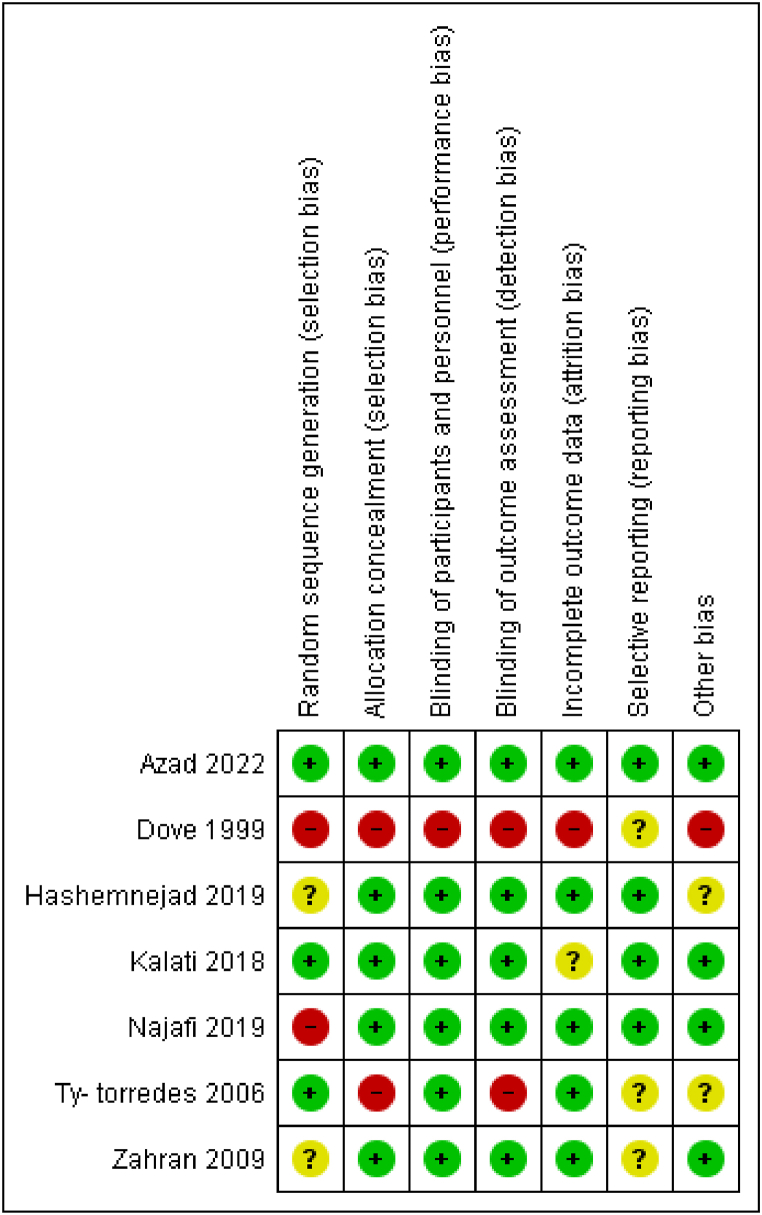
Risk of bias summary of included studies.

Two reviewing authors (SS and MK) independently assessed the risk of bias for each study using seven criteria that are required by Cochrane guidelines for quality assessment of randomized controlled trials (Fig. 2). Three studies reported the method of random sequence generation and were rated as having a low risk for selection bias [19,25,27]. Five studies had a low risk of bias for allocation concealment [10,19,27,28]. Blinding of participants bias was low in six studies and high in one trial [17].Five studies used independent outcome assessors who were blinded to intervention assignment [10,19,[27], [28], [29]]. Only one study was rated as having a high risk for attrition bias [17]. Selective reporting bias was unclear in three trials [17,25,29].

### 3.4 Effects of interventions

#### 3.4.1 Bishop score

**Fig. 3:**
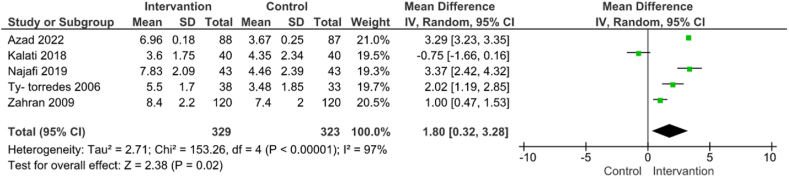
Use of EPO versus placebo on improvement of Bishop score.

The use of EPO for improving Bishop score was evaluated in five studies involving 652 participants [19,25,[27], [28], [29]]. The results showed that EPO could significantly improve Bishop score (MD = 3.23; 95% CI: 3.17, 3.29) (Fig. 3). The level of heterogeneity was high (P < 0.00001; I^2^ = 97%) and did not change using the random effect model, but after utilizing sensitivity analysis and by eliminating three studies [25,27,29], it was reduced to 0% (MD = 3.29; 95% CI: 3.23, 3.35). (Fig. 3).

#### 3.4.2 Length of the second stage of labor

**Fig. 4:**
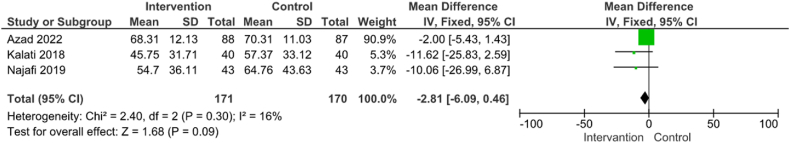
Use of EPO versus placebo on length of the second stage of labor.

EPO was used for assessing the length of the second stage of labor in three studies involving a total of 341 participants [19,27,28]. Results of meta-analysis showed that the difference between two groups regarding length of the second stage of labor was not significant (MD = −2.81; 95% CI: [−6.09, 0.46]) (Fig. 4).

#### 3.4.3 The time interval between EPO administration and birth

**Fig. 5:**
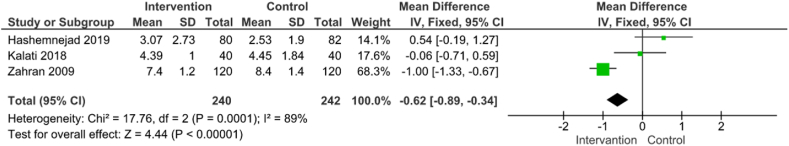
Use of EPO versus placebo on the time interval between primrose administration and birth.

EPO was used for decreasing the interval between its administration and birth in three studies involving 482 participants [10,27,29].There was significant difference between the two studied groups in terms of the time interval between EPO use and childbirth (MD = −0.62; 95% CI: [−0.89, −0.34]) (Fig. 5).

#### 3.4.4 One- and 5-min Apgar scores

**Fig. 6:**
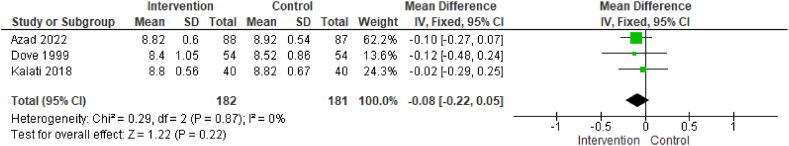
Use of EPO versus placebo on 1-min Apgar score.

Three studies (involving 363 women) reported 1-min Apgar score [17,19,27]. Results of meta-analysis showed no significant effect of EPO on 1-min Apgar score (MD = −0.08; 95% CI: [−0.22, 0.05]) (Fig. 6). Five-minute Apgar score was significantly higher in the EPO group compared to the control groups (MD = 0.48; 95% CI: [0.40, 0.56]) (Fig. 6). After utilizing sensitivity analysis and eliminating one study [17], the level of heterogeneity was reduced to 43%, so the difference between the groups remained significant (MD = 0.65; 95% CI: [0.53, 0.77]). It is worth mentioning that for analyzing Apgar scores at one and 5 min, a study was omitted, because standard deviation was zero in control group, therefore the mean difference was not estimable [28].

**Fig. 7:**
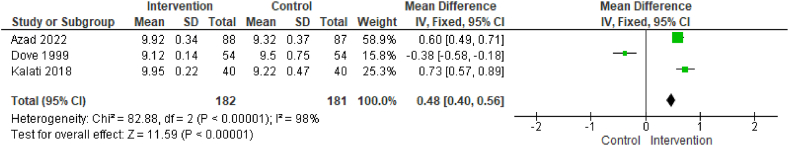
Use of EPO versus placebo on 5-min Apgar score.

With respect to the route of administration, subgroup analysis was performed for five studies containing 652 participants [19,25,[27], [28], [29]]. Three studies, including 391 women, were in the oral administration route subgroup, while two studies including 261 women were in the vaginal administration route subgroup. While vaginal administration of EPO improved bishop score (MD = 3.29; 95% CI: [3.23, 3.35]), a significant difference was observed between the two compared groups in terms of oral administration of EPO (MD = 0.89; 95% CI: [0.49, 1.30]). After utilizing sensitivity analysis and by eliminating one study [27], the level of heterogeneity was reduced to 76%, and the difference between the groups regarding oral administration of EPO remained significant (MD = 1.46; 95% CI: [0.46, 2.45]).

#### 3.4.5 Adverse event reported

Of the seven included studies, two studies reported no adverse events [19,27]. In one study nausea, vomiting, diarrhea and hyperthermia reported in both intervention and control groups [29]. In another study, there were five cases of meconium stained amniotic fluid in the intervention group, and there was one case of asphyxia neonatorum in the control group [25]. One study reported protracted active phase, prolonged rupture of membranes and arrest of descent in intervention group [17]Two studies did not report on adverse events [10,28].

## 4 Discussion

The purpose of this study was to investigate the effect of EPO on cervical ripening and birth outcomes. The results of this systematic review showed that EPO could significantly improve Bishop score. It is believed that gama-linoleic acid in EPO facilitates E2 prostaglandin synthesis and that prostaglandins play the most important role in the process of cervical ripening, as determined by Bishop score [30].

Several studies have indicated the effectiveness of EPO in the improvement of Bishop score during pregnancy, and even before gynecologic procedures [19,26,31,32], but some have not shown any positive effect of this herbal oil on cervical priming of pregnant women [17,27,29].The contradictory results of these studies could be attributed to differences in route or frequency of EPO administration, EPO dosage, initiation time and length of using EPO during pregnancy, complete or incomplete use of EPO capsules by the intervention group and combination of EPO with other medications like misoprostol or oxytocin, which provides greater effect than EPO or these medications alone [11,13,26,30]. Hence, this lack of homogeneity among studies caused that despite performing subgroup analysis, the I square did not decrease significantly. But regarding the discrepancies among studies, most of them reporting a significant difference between the two comparison groups in terms of Bishop score and vaginal route of administration of EPO [10,19,26,28]. However, due to high variation between studies, further clinical trials of high methodological quality are needed to obtain certain results.

In the current study, the latent and active phases of the first stage of labor were not analyzed, because one related article was excluded from the study and the reporting of the two included articles on latent and active phases of labor were different in terms of time. In the present meta-analysis, EPO administration did not decrease the length of the second stage of labor, which is in line with the results of three other trials [19,27,28]. In this regard, Najafi et al. (2019) argued in their study that vaginal use of EPO significantly reduced length of the latent phase in the intervention group, which can be attributed to cervix ripening. They pointed out that since the effect of EPO was related to its half-life and taking EPO by intervention group was stopped at the onset of labor, it showed no effect on the other stages of labor except for the latent phase [28]. In our study, a significant difference was observed between the two groups, in terms of the time interval between taking EPO and childbirth. It is believed that following cervical assessment, the length of time until the onset of labor is directly related to the cervical ripening [17].

There was not any difference between the two groups regarding to 1-min Apgar score, eventhough 5-min Apgar scores were higher among the EPO group. Hemmatzadeh et al. in their systematic review and meta-analysis reported no significant difference between the two comparison groups in terms of one- and 5-min Apgar scores and discussed that they assessed mentioned outcomes in two studies with small sample sizes without enough power [33]. However, we included four studies in our meta-analysis in terms of Apgar scores, but according to GRADE the quality of studies for secondary outcome of Apgar score were low and may lead to unreliable results.

There have been reports indicating that using EPO during pregnancy does not increase complications [13,25]. However, Dove and Johnson concluded that the oral use of EPO in pregnancy, may be associated with increased incidence of premature rupture of membranes, use of oxytocin for augmentation, arrest of descent and higher usage of vacuum [17]. Wedig and Whitsett reported widespread ecchymosis and petechiae in an infant following the mother's use of raspberry leaf tea and EPO during her pregnancy for ripening of the cervix [34]. Hence, for acquiring strong evidence in this regard, more well-designed randomized clinical trials are needed.

### 4.1 Limitations of the study

Because of the high level of heterogeneity among the included studies, results of the present systematic review should be interpreted with caution. Also, all included studies except for two were conducted in developing countries such as Iran and Egypt, which is a challenge in generalizing results. In addition, the limited number of included studies led to decreasing the number of outcomes and detailed analysis.

## 5 Conclusion

This systematic review and meta-analysis showed that the use of EPO during pregnancy significantly improved Bishop score and reduced the length of the time interval between EPO administration and birth. In addition, it was significantly effective in increasing 5-min Apgar score of neonates. Therefore, EPO might offer a natural way for enhancing cervical ripening.

## Author contribution statement

Sholeh Shahinfar, Parvin Abedi, Shayesteh Jahanfar: Conceived and designed the experiments; Performed the experiments; Analyzed and interpreted the data; Contributed reagents, materials, analysis tools or data; Wrote the paper.

Mahin Khajehpoor: Conceived and designed the experiments; Analyzed and interpreted the data; Contributed reagents, materials, analysis tools or data.

Mohammadreza Chashmyazdan: Conceived and designed the experiments; Contributed reagents, materials, analysis tools or data.

## Funding statement

This research did not receive any specific grant from funding agencies in the public, commercial, or not-for-profit sectors.

## Data availability statement

No data was used for the research described in the article.

## Declaration of interest's statement

The authors declare no competing interests.

## Abbreviations

IOL: induction of labor
EPO: evening primrose oil
PROSPERO: Prospective Register of Systematic Reviews
