# Supplementary material for: The effect of evening primrose oil on cervical ripening and birth outcomes: A systematic review and meta-analysis
Source: Heliyon. 2023 Feb 8;9(2):e13414. doi: 10.1016/j.heliyon.2023.e13414 (PMC9947258; doi:10.1016/j.heliyon.2023.e13414)
Supplement: Supplementary file 1 [file mmc1.docx]

Embase Database:

#1 'primrose oil': ab, ti OR 'evening primrose oil': ab,ti OR 'evening primrose':ab,ti OR 'evening-primrose':ab,ti OR epo: ab,ti OR evpo: ab,ti OR oenothera:ab,ti

#2 'bishop score': ab,ti OR 'cervical ripening': ab,ti OR 'ripening of the cervix': ab,ti OR 'ripening of the vagina': ab,ti OR 'vaginal ripening': ab,ti OR labour and delivery: ab,ti OR 'pregnancy outcome': ab,ti OR 'labour stage': ab,ti OR 'labor stage': ab,ti OR 'prolonged pregnancy': ab,ti OR dilatation: ab,ti OR 'cervix preparation': ab,ti OR 'delivery induction': ab,ti OR 'uterine cervix ripening': ab,ti OR 'labor induction': ab,ti OR pregnancy: ab,ti

#3 #1 AND #2

Web of Science:

TS= (“Evening primrose oil” OR “primrose oil” OR “evening primrose” OR “evening-primrose” OR EPO OR EvPO OR Oenothera) AND TS=(“Bishop Score” OR “Cervical Ripening” OR “Ripening of the Cervix” OR “Vaginal Ripening” OR “Ripening of the vagina” OR “labour and delivery” OR “Pregnancy outcome” OR “Prolonged Pregnancy” OR “Labour Stage” OR Dilatation OR “Cervix preparation” OR “Delivery induction” OR “uterine cervix ripening” OR “labor stage” OR “labor induction” OR Pregnancy)

Scopus:

TITLE-ABS-KEY(“Evening primrose oil” OR “primrose oil” OR “evening primrose” OR “evening-primrose” OR EPO OR EvPO OR Oenothera) AND TITLE-ABS-KEY(“Bishop Score” OR “Cervical Ripening” OR “Ripening of the Cervix” OR “Vaginal Ripening” OR “Ripening of the vagina” OR “labour and delivery” OR “Pregnancy outcome” OR “Prolonged Pregnancy” OR “Labour Stage” OR Dilatation OR “Cervix preparation” OR “Delivery induction” OR “uterine cervix ripening” OR “labor stage” OR “labor induction” OR Pregnancy)

Cochran Library:

#1 (“Evening primrose oil” OR “evening primrose” OR “evening-primrose” OR EPO OR EvPO OR Oenothera OR “primrose oil”):ti,ab,kw

#2 (“Bishop Score” OR “Cervical Ripening” OR “Ripening of the Cervix” OR “Vaginal Ripening” OR “Ripening of the vagina” OR “labour and delivery” OR “Pregnancy outcome” OR “Prolonged Pregnancy” OR “Labour Stage” OR Dilatation OR “Cervix preparation” OR “Delivery induction” OR “uterine cervix ripening” OR “labor stage” OR “labor induction” OR Pregnancy):ti,ab,kw

#3 #1 AND #2

Pubmed: 326

(((((((((("Evening primrose oil"[Title/Abstract]) OR (evening primrose oil[MeSH Terms])) OR ("evening primrose"[Title/Abstract])) OR (Oenothera biennis[MeSH Terms])) OR ("evening-primrose"[Title/Abstract])) OR (EPO[Title/Abstract])) OR (EvPO[Title/Abstract])) OR (Oenothera[Title/Abstract])) OR (Oenothera[MeSH Terms])) OR ("primrose oil"[Title/Abstract])) AND (((((((((((("Bishop Score"[Title/Abstract]) OR ("Cervical Ripening"[Title/Abstract])) OR ("Cervical Ripening"[MeSH Terms])) OR ("Ripening of the Cervix"[Title/Abstract] OR "Vaginal Ripening"[Title/Abstract] OR "Ripening of the vagina"[Title/Abstract] OR "labour[Title/Abstract] AND delivery"[Title/Abstract] OR "Pregnancy outcome"[Title/Abstract])) OR ("Pregnancy Outcome"[MeSH Terms])) OR ("Prolonged Pregnancy"[Title/Abstract])) OR ("Pregnancy, Prolonged"[MeSH Terms])) OR ("Labour Stage"[Title/Abstract] OR Dilatation[Title/Abstract] OR "Cervix preparation"[Title/Abstract] OR "Delivery induction"[Title/Abstract] OR "uterine cervix ripening"[Title/Abstract] OR "labor stage"[Title/Abstract])) OR ("labor induction"[Title/Abstract])) OR ("Labor, Induced"[MeSH Terms])) OR (Pregnancy[Title/Abstract])) OR (Pregnancy[MeSH Terms]))

Results

| Embase | 235 |
| --- | --- |
| Web of Science | 189 |
| Scopus | 436 |
| Cochran Library | 94 |
| Pubmed | 326 |
